# New d-dimer threshold for Japanese patients with suspected pulmonary embolism: a retrospective cohort study

**DOI:** 10.1186/s12245-019-0242-y

**Published:** 2019-08-28

**Authors:** Jin Takahashi, Takashi Shiga, Yuita Fukuyama, Yuiko Hoshina, Yosuke Homma, Michiko Mizobe, Kenji Numata, Tetsuya Inoue, Hiraku Funakoshi

**Affiliations:** 1 Department of Emergency and Critical Care Medicine, Tokyo Bay Urayasu Ichikawa Medical Center, 3-4-32 Todaijima, Urayasu, Chiba, 279-0001 Japan; 2 Department of Emergency Medicine, International University of Health and Welfare, 1-4-3 Mita, Minato, Tokyo, 108-8329 Japan; 3 Division of Strategic Planning and Analysis, Tokyo Bay Urayasu Ichikawa Medical Center, 3-4-32 Todaijima, Urayasu, Chiba, 279-0001 Japan

**Keywords:** Pulmonary embolism, New d-dimer threshold, Emergency department, Japan, Factor V Leiden, Prothrombin gene G20210A mutations

## Abstract

**Background:**

In the diagnosis of pulmonary embolism (PE), the d-dimer threshold is based on studies conducted in Western countries, where the incidence rate is 5 times higher than that in Asian countries, including Japan. If we could elevate the d-dimer threshold based on the low pre-test probability in the Japanese population, we could omit the computed tomography pulmonary angiography (CTPA) which might lead to radiation exposure and contrast-induced nephropathy. Therefore, we aimed to determine a new d-dimer threshold specific to Japanese individuals.

**Methods:**

We conducted a retrospective cohort study at an emergency department in Japan, using medical charts collected from January 2013 to July 2017. We included patients whose d-dimer were measured for suspicion of PE with low or intermediate probability of PE and CTPA were performed. The primary outcome was failure rate of the new d-dimer threshold, defined as the rate of PE detected by CTPA among patients with d-dimer under the new threshold ranging from 1000 to 1500 μg/L by 100. The new d-dimer threshold was appropriate if the upper limit of 95% confidence interval of the failure rate of PE was approximately 3%.

**Results:**

In 395 patients included, the number of patients with PE was 24 (the prevalence was 6.1%). If the d-dimer threshold was 1100 μg/L, the failure rate was 0% (0/119), the upper limit of the 95% confidence interval of the failure rate was 3.1%, and 30% (119/395) of the CTPA might be omitted.

**Conclusion:**

The new d-dimer threshold could safely exclude PE. This result can be generalized to other Asian populations with a lower incidence of PE. Further prospective studies will be needed.

## Introduction

Pulmonary embolism (PE) is the third leading cause of death from cardiovascular diseases with an annual incidence of 100–200 per 100,000 population in Europe and the USA [1, 2]. Because the clinical diagnosis of PE is non-specific, the diagnosis of PE should be based on a clinical prediction rule (CPR), d-dimer, and imaging test such as computed tomography pulmonary angiography (CTPA) [1, 3]. Recent research has been published regarding concerns with respect to the potential overuse of diagnostic tests such as d-dimer and CTPA, along with the possible overdiagnosis of PE [3–5].

The d-dimer threshold is generally defined based on studies conducted in Europe and the USA [5, 6]. The incidence of PE in Asian countries, including Japan, is lower than that in Western countries [6–9]. The reasons for the lower incidence of PE in the Asian population are not fully understood but might be caused by genetic and environmental factors, among others [8]. The major thrombophilic defects, the Factor V Leiden and prothrombin gene G20210A mutations, are rare in the Asian population, including Japanese individuals [8, 10, 11]. An elevation in the d-dimer threshold based on the low pre-test probability in this population would allow the omission of the CTPA, a procedure that may lead to the risk of radiation exposure and contrast-induced nephropathy, longer emergency department (ED) stays, higher rates of hospital admission, and excessive medical costs. Indeed, previous research has suggested that ethnicity should be considered as part of the pre-test probability assessment for patients with suspected PE [8]. Therefore, determination of a d-dimer threshold specific to Asian individuals is needed. However, there has been little research evaluating the d-dimer threshold in the Asian population.

To address the knowledge gap on this subject in the current literature, we aimed to determine the new d-dimer threshold specific to the Asian population.

## Methods

### Study design and setting

This study was a retrospective cohort study conducted at a single center, Tokyo Bay Urayasu Ichikawa Medical Center, a 344-bed urban acute care community hospital in Urayasu city, Chiba Prefecture, Japan. The ED in this hospital had a census of 8800 ambulance arrivals and a total of 25,500 visits per year. The study was approved by the institutional review board of the Tokyo Bay Urayasu Ichikawa Medical Center. The requirement for informed consent was waived because the study was retrospective and patient information was anonymized and de-identified prior to analysis.

### Data collection and pressing

**Fig. 1:**
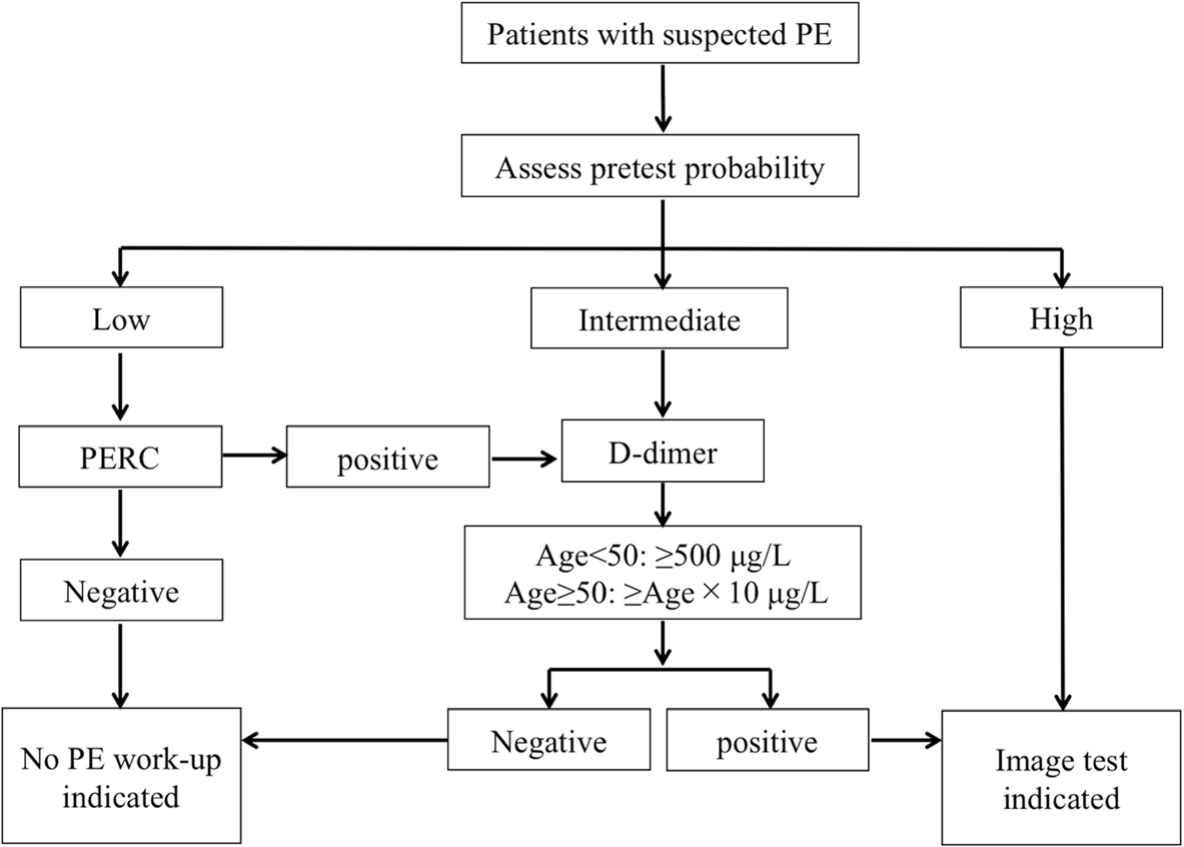
The diagnostic algorithm for pulmonary embolism used in our emergency department. This diagnostic algorithm consists of the clinical prediction rule and d-dimer, in which incorporated pulmonary embolism rule-out criteria (PERC) and age-adjusted d-dimer cut-off rule. Abbreviation: PE pulmonary embolism, PERC pulmonary embolism rule-out criteria

We briefly describe the method of diagnosis of PE in our department. Since 2014, our department has used a CPR that incorporated the pulmonary embolism rule-out criteria (PERC) rule and an age-adjusted d-dimer cut-off rule for the diagnosis of PE (Fig. 1) [4, 5, 12, 13]. This algorithm is the same as that recommended by the clinical guidelines of the American College of Physicians in 2015 [3]. If the probability of PE in the CPR is low, the PERC rule is considered. If none of the eight items in the PERC rule is present, PE can be excluded without measuring d-dimer. If more than one of the eight items among those in the PERC rule, d-dimer is measured. Even in cases in which the probability of PE according to the CPR is intermediate, d-dimer is measured. To determine whether the d-dimer is positive or negative, the age-adjusted rule is used. If the d-dimer is < age × 10 μg/L in age ≥ 50 years, or if the d-dimer is < 500 μg/L in age < 50 years, the d-dimer is considered negative, and PE can be excluded. If the d-dimer is positive (i.e., the d-dimer is ≥ age × 10 μg/L in age ≥ 50 years, or the d-dimer is ≥ 500 μg/L in age < 50 years), or if the possibility of PE is high in the CPR, CTPA is performed.

VIDAS d-dimer Exclusion II (BioMérieux, Marcy-L’etoile, France) high-sensitive d-dimer assay was used in our department. The 320-row area detector CT (Aquilion ONE; Canon Medical Systems, Yokohama, Japan) or 64-row detector CT (Aquilion CXL; Canon Medical Systems) was used for CTPA. All CTPA procedures performed in our department were reviewed by board-certified radiologists.

### Selection of participants

We reviewed patient medical records from between January 2013 to July 2017. We included consecutive adults (age ≥ 18 years) who met the following criteria: (1) d-dimer was measured for suspicion of PE with low or intermediate probability in the ED and (2) value of d-dimer > 500 μg/L in patients ≤ 50 years old, or value of d-dimer > age × 10 μg/L in patients > 50 years old. Based on previous studies [5, 14, 15], we excluded patients based on the following criteria: (1) pregnancy, (2) non-Japanese patients, (3) medication with oral anticoagulants, (4) contraindications to contrast medium (allergy to contrast medium, uncontrolled hyperthyroidism, or biguanide diabetes medicine), (5) impaired renal function (creatinine clearance less than 30 mL/min), (6) life expectancy of less than 3 months, (7) patient refusal or lack of administration of CTPA, or (8) missing data (age, sex, or component factors of the CPR).

In 2013 and 2014, because this period was prior to induction of the age-adjusted d-dimer cut-off rule in our department, CTPA was performed in patients > 50 years old whose d-dimer was > 500 μg/L. During this period, in patients aged > 50 years, we included patients with d-dimer > age × 10 μg/L and excluded patients with d-dimer ranging from 500 to age × 10 μg/L, even if CTPA was performed.

### Outcome measure

The outcome measure of interest was the failure rate of the new d-dimer threshold, defined as the rate of PE detected by CTPA among the patients with d-dimer under the new threshold.

### Statistical analysis

Because d-dimer of the patients included in this study was predicted to tend toward higher levels than the age-adjusted value of d-dimer, we established a new d-dimer threshold increasing from 1000 to 1500 μg/L by 100, and we calculated the failure rate of PE at each threshold. Based on the results of previous studies, if the upper limit of the 95% confidence interval (CI) of the failure rate of PE is around 3%, we assessed that the new d-dimer threshold is appropriate [5, 14–16].

Baseline characteristics of groups with or without PE were expressed as number (%) for categorical variables and median (interquartile range [IQR]) for continuous variables. Categorical variables were compared using Fisher’s exact test, and continuous variables were compared using the Wilcoxon rank-sum test. *P* <  0.05 was considered statistically significant. Analyses were performed with the use of JMP 14.0.0 (SAS Institute, Inc., Cary, NC, USA).

## Results

**Fig. 2:**
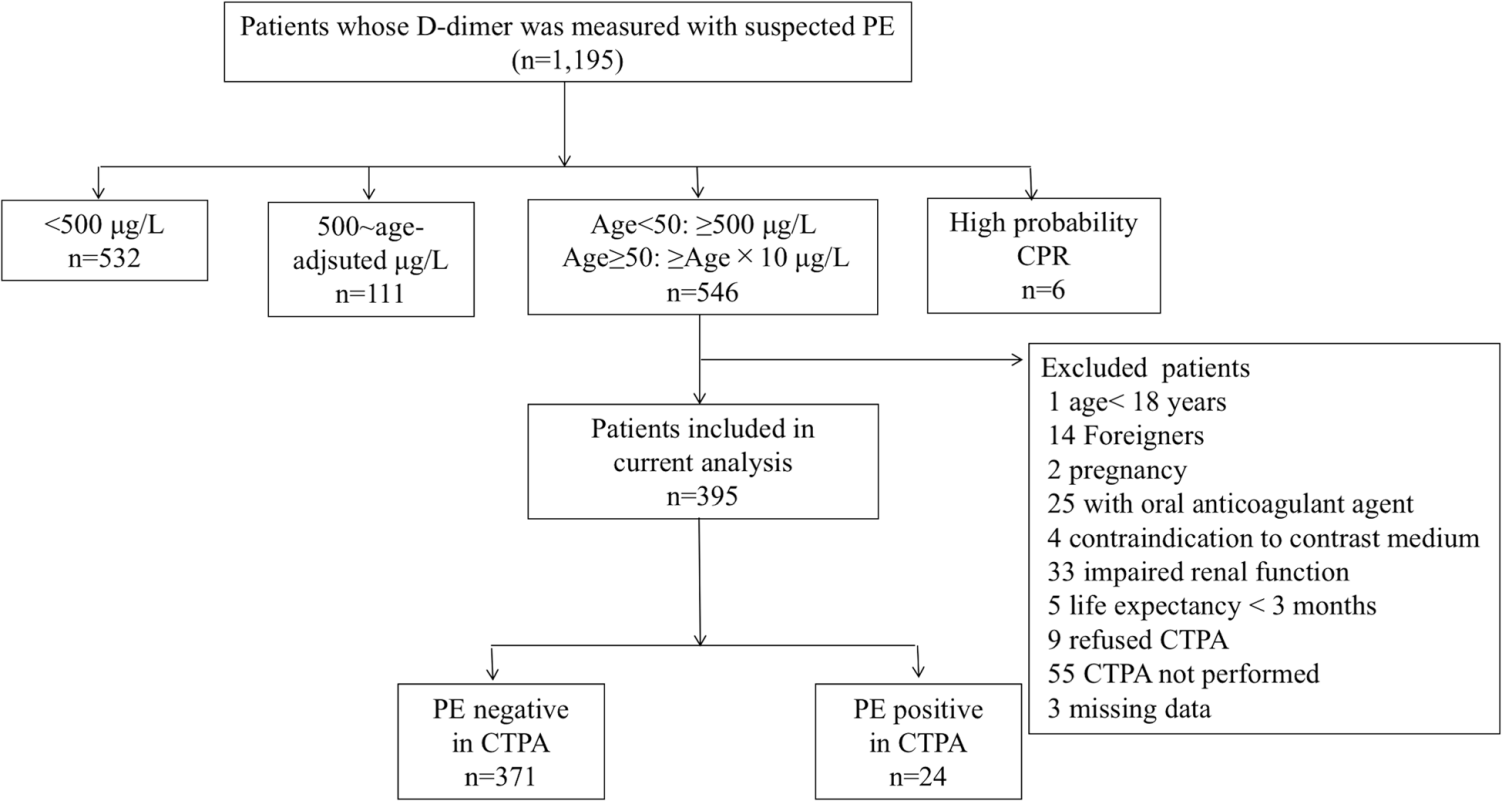
Patients suspected pulmonary embolism in our emergency department. 1195 patients suspected PE and measured d-dimer in our department. Of these, 395 patients were eligible for the current analysis. Abbreviation: PE, pulmonary embolism; CPR, clinical prediction rule; CTPA, computed tomography pulmonary angiography

During the 55-month study period, we analyzed the records of 1195 patients with suspected PE and measured d-dimer in our department. Of these, 546 met the inclusion criteria. We excluded 151 patients based on the criteria described previously. After application of the study criteria, 395 patients were eligible for the analysis Fig. 2. Among these patients, the number of patients with PE was 24 (i.e., the prevalence was 6.1%). Overall, the median age was 75 years (IQR, 63–84 years) and 47% were men. The median value of d-dimer was 1523 μg/L (IQR, 1019–3564 μg/L). Characteristics of patients in the PE-negative and PE-positive groups are shown in Table 1. The patients with PE were younger and had significantly higher Wells score and d-dimer values compared with patients without PE. The median value (IQR) of the pulmonary embolism severity index was 96 points (IQR, 74–116 points).

**Table 1:** Characteristics of patients whose d-dimer was measured and computed tomography pulmonary angiography was performed, according to pulmonary embolism

	PE negative *n* = 371	PE positive *n* = 24	*P* value^†^
Age, median (IQR), years	75 (63–84)	67 (49–76)	0.02
Male sex	163 (44)	13 (54)	0.40
Malignancy^a^	56 (15)	3 (13)	1.00
Prior PE or DVT	8 (2)	1 (4)	0.43
Hormone use	7 (2)	0 (0)	1.00
Wells score, median (IQR)	0 (0–1.5)	1.5 (0–3)	< 0.001
d-dimer value, median (IQR), μg/L	1455 (976–3345)	5035 (2025–8815)	0.001

*Abbreviations*: *PE* pulmonary embolism, *DVT* deep vein thrombosis, *IQR* interquartile range

Data are shown as *n* (%) unless otherwise specified

^a^Cancer treatment currently or in last 6 months or receiving palliative care

†Wilcoxon rank-sum test for continuous variables. Fisher’s exact test for categorical variables

As the primary outcome, we demonstrated the failure rate (95% CI) of the new d-dimer threshold, which ranged from 1000 to 1500 μg/L (Table 2). When the new d-dimer threshold was set to 1100 μg/L, the failure rate was 0%, and the upper limit of the 95% CI of the failure rate was 3.1%. If the threshold of 1100 μg/L is used, 30% (119/395) of the CTPA might be omitted. Other thresholds were not appropriate because the upper limit of the 95% CI of the failure rate for each threshold was not close to 3%.

**Table 2:** Number of pulmonary embolisms, failure rate, and 95% confidence interval, according to new d-dimer threshold

New d-dimer threshold μg/L	Included patients^a^ n	No. of PEs n	Failure rate (95% CI) %
< 1000	97	0	0 (0–3.8)
< 1100	119	0	0 (0–3.1)
< 1200	144	1	0.7 (0.1–3.8)
< 1300	164	3	1.8 (0.6–5.2)
< 1400	183	5	2.7 (1.2–6.2)
< 1500	198	5	2.5 (1.1–5.8)

*Abbreviation*: *PE* pulmonary embolism, *CI* confidence interval

^a^Patients with d-dimer under new threshold

## Discussion

Our findings have revealed that the new d-dimer threshold (i.e., 1100 μg/L) could safely exclude PE. To our knowledge, this is the first study to demonstrate a new d-dimer threshold specific to the Japanese population.

Several studies with the aim of elevating the d-dimer threshold for the purpose of reducing unnecessary CTPA procedures have been conducted. Because d-dimer levels increase with age, false-positive results of d-dimer testing also increase, and as a result, the unnecessary performance of CTPA is increasing. In a previous study, this difficulty was partly resolved through introduction of a threshold of age × 10 [5]. In another study, patients were managed using different d-dimer levels and a CPR of three items (clinical signs of deep vein thrombosis, hemoptysis, and whether PE is the most likely diagnosis) [17]. In patients with no items and d-dimer less than 1000 μg/L, or in patients with one or more items and d-dimer less than 500 μg/L, PE was excluded. Introduction of this assessment led to a reduction in the number of CTPA procedures in comparison with the existing threshold of 500 μg/L. In contrast to the previous studies, our results allowed determination of a higher threshold of d-dimer that could omit the unnecessary performance of CTPA. In addition, compared with former research, we could reduce the performance of CTPA in younger patients who are susceptible to radiation exposure. Furthermore, compared with the latter study, we could simplify the d-dimer threshold. As mentioned in the latter study, a simplified algorithm is important because the simplified algorithm improves adherence to the algorithm and decreases the number of diagnostic failures [17].

In Japan, the incidence of PE demonstrated an increase of more than five times its 1996 level by 2011, owing to the westernization of the lifestyle, increasing use of oral contraceptives, increasing levels of obesity and cancer, and greater recognition of the risk of PE [7]. Nevertheless, the incidence of PE in Japan is about one fifth of that in Western countries [6, 7]. Similarly, the incidence of PE in Asia is lower than that in Western countries [8, 9]. The reasons for this lower incidence of PE might be multifactorial, including genetic and environmental factors [8]. Factor V Leiden and prothrombin gene G20210A gene mutations are representative genetic factors; the frequencies of these are 4.4% and 3.1%, respectively, in European populations [9]. In contrast, Factor V Leiden and prothrombin gene G20210A gene mutations have never been found in Japanese individuals and are rare in the Asian population [9, 18].

Our findings have several implications for the diagnosis of PE. For clinicians, our data might support a strategy of increasing the d-dimer threshold when diagnosing PE in Asian patients, in whom the incidence of PE is low. This strategy may lead to the omission of unnecessary CTPA followed by the lower risk of radiation exposure and contrast-induced nephropathy, shorter ED stays, lower rates of hospital admissions, and reduction of excessive medical costs. For researchers, our data would facilitate prospective and multicenter studies to evaluate the appropriate diagnostic strategy specific to the Asian population.

### Potential limitations

This study has several possible limitations. First, this study was a single-center study, which might lead to low generalizability. Second, despite the finding that the value of d-dimer was applicable to the CTPA, several patients did not undergo CTPA. In post hoc observation, among these patients, none of the hospitalized patients had a diagnosis of PE. Similarly, with the exception of one patient, none of the outpatients among patients who did not undergo CTPA visited again with symptoms of PE. One exception was a 74-year-old male with a d-dimer level of 3705 μg/L. He and his family refused CTPA because he had mild renal failure that might be exacerbated by contrast-induced nephropathy. He was transferred to another hospital where he underwent CTPA and received a diagnosis of PE. Because the value of d-dimer in this patient was over 1100 μg/L, the new d-dimer threshold of 1100 μg/L remains valid. However, among the patients who did not undergo CTPA, some patients might have asymptomatic PE. In addition, some outpatients might be diagnosed with PE at other hospitals, although we instructed them to visit our hospital again when they had symptoms of PE. Therefore, multicenter and multi-national prospective studies will be needed. Third, although all eligible patients underwent CTPA in the initial evaluation in the ED, they did not necessarily undergo follow-up within a definitive period, such as 3 months. A previous study reported that the sensitivity of multidetector CTPA was 83% [19]. This result may indicate that CTPA alone may miss PE. However, in patients with low or intermediate clinical probability and a negative multidirector CTPA result during initial evaluation, the 3-month risk of PE was low (1.5%, 95% CI 0.8–3.0) [14]. Because the probability of PE in the eligible patients in our study was low or intermediate, this limitation might be addressed.

## Conclusions

In this study conducted in Japan, we demonstrated that the new d-dimer threshold (i.e., 1100 μg/L) might safely exclude PE. This result has generalizability to other Asian individuals, who tend to have a lower incidence of PE compared with those in Western countries. Further prospective validation studies conducted in other Asian countries will be needed.

## Data Availability

The datasets generated and analyzed during the current study are not publicly available due personal information management but are possibly available from the corresponding author on reasonable request.

## Abbreviations

CI: Confidence interval
CPR: Clinical prediction rule
CTPA: Computed tomography pulmonary angiography
DVT: Deep vein thrombosis
ED: Emergency department
IQR: Interquartile range
PE: Pulmonary embolism
PERC: Pulmonary embolism rule-out criteria
